# Mortality by occupation and industry among Japanese men in the 2015 fiscal year

**DOI:** 10.1186/s12199-020-00876-3

**Published:** 2020-08-05

**Authors:** Hirokazu Tanaka, Taketo Tanaka, Koji Wada

**Affiliations:** 1 Department of Public Health, Erasmus University Medical Center, Rotterdam, The Netherlands; 2 Department of Public Health, Graduate School of Medicine, The University of Tokyo, Tokyo, Japan; 3 Graduate School of Medicine, International University of Health and Welfare, 26-1 Akasaka-4chome Minato-ku, Tokyo, 107-8402 Japan

**Keywords:** Epidemiology, Cause of Death/trends, Censuses, Socioeconomic Factors, Registries, Middle Aged

## Abstract

**Background:**

Although previous studies have underscored some unique inequalities in occupational mortality in Japan, many of these trends have been dramatically altered during recent decades. We analyzed mortality data by occupation and industry in Japan, to determine whether differences remained by the mid-2010s for men in working-age population.

**Methods:**

We calculated age-standardized all-cause and cause-specific mortality, according to occupation and industry, among men aged 25–64 years in the 2015 fiscal year (1 April 2015 to 31 March 2016). Occupational and industry-specific categories were defined using the Japan Standard Occupational Classification and Japan Standard Industrial Classification, respectively. Age-standardized mortality rates were computed using 5-year age intervals. Mortality rate ratios adjusted for age and 95% confidence intervals (CIs) were estimated using Poisson regression. Cause-specific deaths were classified into four broad groups (cancers [C00-D48], cardiovascular diseases [I00-I99], external causes [V01-Y98], and all other diseases) based on the International Statistical Classification of Diseases 10th Revision (ICD-10).

**Results:**

Clear mortality differences were identified by both occupation and industry among Japanese males. All-cause mortality ranged from 53.7 (clerical workers) to 240.3 (service workers) per 100,000 population for occupation and from 54.3 (workers in education) to 1169.4 (workers in mining) for industry. In relative terms, service workers and agriculture, forestry, and fishing workers had 2.89 and 2.50 times higher all-cause mortality than sales workers. Administrative and managerial workers displayed higher mortality risk (1.86; 95% CI 1.76–1.97) than sales workers. Similar patterns of broad cause-specific mortality inequality were identified in terms of both absolute and relative measures, and all broad cause-specific deaths contributed to the differences in mortality by occupation and industry.

**Conclusions:**

Substantial differences in mortality among Japanese male workers, according to occupation and industry, were still present in 2015.

## Introduction

Japan’s rapidly shrinking working-age population (men and women aged 15–64 years) requires a reorganization of industry and reform of the current working-style, a situation which has involved lifetime commitment to a particular job. Many Japanese, particularly males, have traditionally worked for the same company or firm once they graduated from high school or university. Owing to the unique “Confucian” welfare background and labor protection policies, Japanese society is relatively more tolerant of stricter work ethics and overtime work [1, 2]. When compared internationally, long working hours are still more common among Japanese workers when compared to Western countries and long working hours is suggested to be health risk via poor-health behavior [3]. Given that high occupational burdens may lead to death from overwork (*karoshi*) [4, 5], the importance that occupational health practitioners can play in modern Japanese society cannot be underestimated [6].

Although in Western countries mortality inequality according to occupational class is usually observed as a clear social gradient [7–9], trends are remarkably different in Japan. When compared with European countries, for example, non-manual workers with higher socioeconomic status (e.g., managers and professionals) have higher mortality than non-manual (e.g., clerical, service, sales workers) and manual workers (e.g., craft and related trade workers, semi-skilled and unskilled manual workers), which is defined as lower socioeconomic status [10]. Inequality trends in occupational mortality have changed dramatically during recent decades, with some studies finding that mortality among managers and professionals increased rapidly after the year 2000, in tandem with a restructuring of the Japanese economy during that time [11, 12]. Mortality among clerical and manufacturing process workers decreased steadily during this period, however, ensuring that it is important to monitor differences in mortality by occupation and industry in Japan.

Some previous studies have examined occupational mortality inequality in Japan prior to 2010, finding that male all-cause mortality rate ratios were higher among managers (3.08) and professionals (1.70) compared with manufacturing workers aged 30 to 59 years in 2010 [13]. Regarding mortality owing to lung cancer, service workers (3.28) and managers (3.18) had higher mortality rate than that of male manufacturing workers aged 25 to 64 years [14]. These workers may not have sufficient time to rest and receive health checkups owing to heavy work demands and long working hours. Suicide prevention currently represents a serious health topic in Japan, especially among men of working age. There are also clear differences in suicide rates by occupation and industry. In 2010, for example, Japanese managers, service workers, and farmers had a higher relative risk of suicide (3.91, 3.63, and 3.53, respectively) than sales workers [15]. These trend studies underscore the unique mortality inequalities that existed in Japan prior to 2010. Within this context, the aim of the present study was to reveal whether mortality inequalities among men, according to occupation and industry, persisted through the mid-2010s.

## Methods

### Data sources

National Vital Statistics and occupational and industrial data were analyzed in this study, information that is officially reported every 5 years in the “Report of Vital Statistics: Occupational and Industrial Aspects.” The Ministry of Health, Labour and Welfare (MHLW) of Japan has conducted this survey every 5 years since 1970, coinciding with years of the Population Census [16]. We obtained government records from the National Vital Statistics (death records) of Japan in fiscal year 2015 (from 1 April 2015 to 31 March 2016). We also obtained age- and sex-aggregated population census data for occupation-specific populations (denominator; mid-year population estimates) from the Japanese Population Census, conducted on 1 October 2015 [17]. Our study group has previously reported all-cause and cause-specific mortality according to occupation and industry with regard to trends prior to 2010 [11–15]. Therefore, in this study, we focused on the most recent year with available data.

### Occupations and industries

Occupational categories were defined using the Japan Standard Occupational Classification (11 categories: administrative and managerial; professional; clerical; sales; services; security; agriculture, forestry, and fishing; manufacturing; transport; construction and mining; and carrying, cleaning, and packaging). Industry categories were defined using the 19 categories of the Japan Standard Industrial Classification (agriculture, fishing, mining, construction, manufacturing, electricity and gas, information, transport, wholesale and retail, finance, real estate and rental, research and professional services, accommodation and dining services, entertainment services, education, medical and welfare, combined services, other service industries, and government). These categories roughly correspond to the International Standard Classification of Occupations and International Standard Industrial Classification [18–21].

### Analysis

We calculated age-standardized, all-cause and broad cause-specific mortality for all Japanese men aged 25–64 years according to occupation and industry. Underlying causes of death were classified according to the International Statistical Classification of Diseases 10th Revision (ICD-10) and grouped into four broad groups (cancers [C00-D48], cardiovascular diseases [I00-I99], external causes (including injuries and suicide) [V01-Y98], and all other diseases). Age-standardized mortality rates were directly computed using 1985 standard Japanese population data in 5-year age intervals, which is generally used for standardization for official Japanese statistics. To assess relative measures of mortality inequality, mortality rate ratios adjusted for age and 95% confidence intervals (CIs) were estimated with Poisson regression. We used sales workers (occupation) and wholesale and retail (industry) as reference, respectively. We restricted the analysis to men because previous studies have suggested that differences in mortality are more remarkable among men than women [13]. Stata version 15/SE (StataCorp, College Station, TX, USA) was used for the analyses, and *p* values less than 0.05 were considered statistically significant.

## Results

### Mortality according to occupation

Table 1 displays all-cause and broad cause-specific age-standardized mortality rates by occupation, in addition to the number of workers and deaths among Japanese men of working age, with occupations sorted by ascending order of mortality. A total of 28,695 deaths occurred among male workers during the Japanese fiscal year of 2015. The age-standardized all-cause mortality rate among male workers aged 25 to 64 years was 101.0 per 100,000 population. Broad cause-specific age-standardized mortality rates were as follows: 36.1 (all cancer), 26.8 (cardiovascular disease), 25.2 (external causes), and 12.9 (other causes) per 100,000 population. All-cause mortality among Japanese men was about 7.6 times higher among those who were unemployed when compared to their employed counterparts.

**Table 1:** Age-standardized mortality rates by occupation in Japan during the 2015 fiscal year (per 100,000 population, males aged 25–64 years (among workers aged 25–64 years in the National Census))

	Workers		Deaths	All-cause	Broad, cause-specific death			
	***n***	%	***n***		All cancer (C00-D48)	Cardiovascular disease (I00-I99)	External causes (V01-Y98)	Other causes
**Workers (total)**	24,863,422		28,695	101.0	36.1	26.8	25.2	12.9
**Occupation (JSOC)**								
**Clerical workers**	4,003,816	16.1	2495	53.7	21.1	12.8	13.8	5.9
**Carrying, cleaning, packaging, and related workers**	1,491,304	6.0	1064	62.1	18.5	21.4	14.1	8.1
**Manufacturing process workers**	4,528,220	18.2	3424	73.8	26.2	20.5	18.7	8.4
**Sales workers**	3,377,013	13.6	2890	82.1	32.0	21.9	17.2	10.9
**Security official**	791,484	3.2	794	90.1	27.2	25.3	26.9	10.6
**Transport and machine operating workers**	1,568,689	6.3	2015	100.3	33.5	27.8	27.4	11.6
**Professional workers**	4,173,468	16.8	4840	110.3	43.4	28.6	25.5	12.7
**Construction and mining workers**	2,024,420	8.1	3546	146.9	47.7	37.5	40.6	21.1
**Administrative and managerial workers**	794,143	3.2	2031	193.0	64.6	42.5	62.8	23.1
**Agriculture forestry and fishery workers**	642,983	2.6	2017	211.4	53.8	51.3	72.1	34.1
**Service workers**	1,467,882	5.9	3579	240.3	80.2	66.7	57.9	35.5
**Unemployment**^□^	6,199,344		58,569	765.8	240.0	175.1	137.7	213.0

*JSOC* Japan Standard Occupational Classification

^□^Unemployment includes “Unemployed” and “Population not in the labor force,” as indicated in the Population Census

Manufacturing process workers (18.2%), professional workers (16.8%), clerical workers (16.1%), and sales workers (13.6%) comprised the majority of male Japanese workers. All-cause mortality ranged from 53.7 per 100,000 population (clerical workers) to 240.3 per 100,000 population (service workers). The two groups of “administrative and managerial workers” and “agriculture, forestry, and fishing workers” displayed high mortality rates from external causes (including suicide), with their mortality rates being 62.8 and 72.1 per 100,000 population, respectively. However, we found relatively low mortality rates among clerical workers (53.7 per 100,000 population); carrying, cleaning, packaging, and related workers (62.1 per 100,000 population); manufacturing process workers (73.8 per 100,000 population); and sales workers (82.1 per 100,000 population).

All cancer mortality ranged from 18.5 per 100,000 population (carrying, cleaning, packaging, and related workers) to 80.2 per 100,000 population (service workers), whereas cardiovascular disease mortality ranged from 12.8 per 100,000 population (carrying, cleaning, packaging, and related workers) to 66.7 per 100,000 population (service workers). Agriculture, forestry, and fishing workers had the highest mortality from external causes (72.1 per 100,000 population). We observed similar patterns of broad, cause-specific mortality inequality among workers in these groups.

In relative terms, service workers and agriculture, forestry, and fishing workers had 2.89 (95% CI 2.75–3.03) and 2.50 (95% CI 2.36–2.65) times higher all-cause mortality when compared with sales workers (Table 2). Administrative and managerial workers had higher mortality risk (1.86; 95% CI 1.76–1.97) when compared with sales workers. We also observed similar patterns of broad cause-specific mortality inequality in terms of relative measures among workers in these groups. We observed similar patterns of broad, cause-specific mortality inequality among workers in these groups.

**Table 2:** Mortality rate ratios by occupation in Japan during the 2015 fiscal year, males aged 25–64 years

	All-cause		Broad, cause-specific death							
			All cancer (C00-D48)		Cardiovascular disease (I00-I99)		External causes (V01-Y98)		Other causes	
	RR	95% CI	RR	95% CI	RR	95% CI	RR	95% CI	RR	95% CI
**Occupation (JSOC)**										
**Clerical workers**	0.64	0.61–0.68	0.66	0.60–0.71	0.58	0.52–0.65	0.77	0.69–0.87	0.54	0.47–0.63
**Carrying, cleaning, packaging, and related workers**	0.73	0.68–0.78	0.55	0.49–0.62	0.90	0.79–1.02	0.84	0.72–0.98	0.70	0.58–0.85
**Manufacturing process workers**	0.90	0.85–0.94	0.81	0.75–0.88	0.92	0.84–1.01	1.09	0.98–1.21	0.77	0.67–0.89
**Sales workers**	Reference		Reference		Reference		Reference		Reference	
**Security official**	1.10	1.02–1.19	0.83	0.73–0.95	1.17	1.02–1.36	1.57	1.34–1.83	0.98	0.78–1.23
**Transport and machine operating workers**	1.18	1.12–1.25	1.02	0.93–1.12	1.24	1.11–1.38	1.51	1.33–1.71	1.06	0.91–1.25
**Professional workers**	1.35	1.29–1.42	1.37	1.27–1.47	1.31	1.19–1.43	1.51	1.36–1.67	1.17	1.03–1.34
**Construction and mining workers**	1.79	1.70–1.88	1.51	1.39–1.63	1.68	1.53–1.85	2.36	2.12–2.63	1.90	1.66–2.17
**Administrative and managerial workers**	1.86	1.76–1.97	1.89	1.73–2.05	1.67	1.50–1.87	2.07	1.80–2.38	1.58	1.34–1.86
**Agriculture forestry and fishery workers**	2.50	2.36–2.65	1.79	1.62–1.96	2.18	1.95–2.44	4.32	3.83–4.87	2.97	2.56–3.44
**Service workers**	2.89	2.75–3.03	2.43	2.24–2.64	2.92	2.66–3.20	3.33	3.00–3.70	3.16	2.77–3.61

*JSOC* Japan Standard Occupational Classification, *RR* rate ratio, *95% CI* 95% confidence interval

### Mortality according to industry

Table 3 shows all-cause and broad cause-specific age-standardized mortality rates, by occupation. Manufacturing (22.4%), wholesale and retail (13.4%), construction (11.8%), and transport (8.3%) comprised the largest share of industry sectors. There were very few workers in fishing and mining. Male Japanese workers in the education sector had the lowest mortality (54.3 per 100,000 population) whereas those in the mining sector had the highest mortality (1169.4 per 100,000 population). We found a clear difference in all-cause and broad cause-specific mortality for education and accommodation and dining services; however, the difference was steep for agriculture and forestry (222.2 per 100,000 population) and mining (1169.4 per 100,000 population). We observed similar patterns of broad, cause-specific mortality inequality among workers in these groups.

**Table 3:** Age-standardized mortality rates by industry in Japan during the 2015 fiscal year (per 100,000 population, males aged 25–64 years (among workers aged 25–64 years in the National Census))

	Workers		Deaths	All-cause	Broad, cause-specific death			
	***n***	%	***n***		All cancer (C00-D48)	Cardiovascular disease (I00-I99)	External causes (V01-Y98)	Other causes
**Industry (JSIC)**								
**Education**	931,896	3.8	621	54.3	20.4	14.5	13.6	5.7
**Wholesale and retail**	3,324,487	13.4	2479	67.4	26.4	18.2	13.9	8.9
**Combined services**	268,198	1.1	205	69.0	30.6	18.3	14.4	5.7
**Research and professional services**	1,023,116	4.1	903	75.0	30.0	17.4	17.9	9.6
**Real estate and rental**	486,777	2.0	477	77.8	29.8	18.1	18.1	11.7
**Manufacturing**	5,566,866	22.4	4688	79.6	30.2	20.7	19.3	9.4
**Medical and welfare**	1,378,167	5.6	1201	85.0	29.9	22.0	21.9	11.1
**Government**	1,308,767	5.3	1230	86.6	33.5	19.8	25.1	8.2
**Finance**	576,360	2.3	641	93.6	39.6	22.0	20.8	11.3
**Other services**	1,587,656	6.4	1902	96.9	29.1	31.8	25.3	10.7
**Information**	1,124,217	4.5	1024	103.9	39.3	27.8	22.7	14.0
**Transport**	2,058,529	8.3	2635	104.8	36.2	29.5	27.2	11.8
**Entertainment services**	577,575	2.3	739	121.3	40.6	35.4	28.7	16.6
**Construction**	2,922,169	11.8	4438	123.3	42.1	31.3	33.6	16.3
**Accommodations and dining services**	787,440	3.2	1296	151.2	44.4	41.8	37.9	27.1
**Agriculture and forestry**	576,079	2.3	1905	222.2	57.9	55.0	75.9	33.5
**Electricity and gas**	220,095	0.9	727	297.6	106.0	73.5	85.9	32.1
**Fisheries**	73,280	0.3	388	417.4	127.3	97.7	121.4	71.0
**Mining**	15,474	0.1	232	1169.4	460.0	269.3	316.8	123.4

*JSIC* Japan Standard Industrial Classification

Table 4 indicates mortality rate ratios according to industry among working-age Japanese males. We found a relatively moderate mortality difference for entertainment services (1.77; 95% CI 1.63–1.92) and the construction sector (1.81; 95% CI 1.73–1.90), as compared with wholesale and retail workers. However, mining (16.6; 95% CI 14.5–19.0) and fishing (5.52; 95% CI 4.96–6.14) had quite high mortality rate ratios, in comparison with wholesale and retail workers.

**Table 4:** Mortality rate ratios by industry in Japan during the 2015 fiscal year, males aged 25–64 years

	All-cause		Broad, cause-specific death							
			All cancer (C00-D48)		Cardiovascular disease (I00-I99)		External causes (V01-Y98)		Other causes	
	RR	95% CI	RR	95% CI	RR	95% CI	RR	95% CI	RR	95% CI
**Industry (JSIC)**										
**Education**	0.79	0.72–0.86	0.75	0.66–0.86	0.79	0.67–0.94	0.97	0.80–1.18	0.64	0.49–0.83
**Wholesale and retail**	Reference		Reference		Reference		Reference		Reference	
**Combined services**	1.02	0.88–1.17	1.14	0.93–1.42	1.03	0.78–1.35	1.02	0.74–1.42	0.65	0.40–1.05
**Real estate and rental**	1.10	1.00–1.22	1.04	0.90–1.21	0.94	0.77–1.15	1.25	0.99–1.57	1.30	1.01–1.67
**Research and professional services**	1.10	1.02–1.18	1.12	0.99–1.25	0.96	0.83–1.12	1.26	1.06–1.49	1.06	0.85–1.31
**Manufacturing**	1.16	1.11–1.22	1.14	1.05–1.23	1.14	1.04–1.25	1.37	1.23–1.52	1.05	0.92–1.21
**Medical and welfare**	1.25	1.16–1.33	1.10	0.99–1.24	1.19	1.04–1.36	1.56	1.35–1.80	1.25	1.03–1.51
**Government**	1.31	1.22–1.40	1.33	1.19–1.48	1.13	0.99–1.30	1.81	1.58–2.09	0.97	0.78–1.19
**Other services**	1.37	1.29–1.46	1.03	0.93–1.13	1.69	1.52–1.88	1.77	1.55–2.02	1.20	1.01–1.43
**Finance**	1.38	1.27–1.51	1.52	1.34–1.73	1.21	1.01–1.44	1.45	1.19–1.77	1.27	0.99–1.63
**Information**	1.45	1.35–1.56	1.42	1.26–1.60	1.51	1.31–1.73	1.54	1.32–1.80	1.50	1.23–1.84
**Transport**	1.54	1.45–1.62	1.36	1.25–1.49	1.63	1.47–1.81	1.94	1.71–2.19	1.35	1.16–1.58
**Entertainment services**	1.77	1.63–1.92	1.49	1.30–1.71	1.89	1.63–2.21	2.02	1.69–2.41	1.88	1.51–2.35
**Construction**	1.81	1.73–1.90	1.61	1.49–1.74	1.72	1.57–1.90	2.39	2.14–2.67	1.80	1.57–2.06
**Accommodations and dining services**	2.20	2.05–2.35	1.67	1.49–1.88	2.24	1.97–2.55	2.62	2.27–3.03	3.02	2.55–3.57
**Agriculture and forestry**	3.13	2.95–3.32	2.27	2.06–2.51	2.73	2.42–3.07	5.48	4.82–6.23	3.69	3.15–4.32
**Electricity and gas**	4.26	3.92–4.63	3.96	3.46–4.53	4.15	3.54–4.88	5.87	4.96–6.96	3.54	2.77–4.53
**Fisheries**	5.52	4.96–6.14	4.25	3.56–5.08	4.90	3.95–6.08	8.66	6.93–10.82	6.65	5.07–8.73
**Mining**	16.6	14.5–19.0	17.0	13.9–20.7	13.3	9.99–17.7	21.7	16.2–29.2	14.6	9.86–21.6

*JSIC* Japan Standard Industrial Classification, *RR* rate ratio, *95% CI* 95% confidence interval

## Discussion

Overall, this study revealed that clear differences in mortality rates by occupation persisted during 2015 in Japan. Mortality rates among administrative and managerial workers and professional workers (commonly described as high-grade occupational classes) remained relatively high in 2015, a finding which is consistent with previous research. Japanese managers and professionals have experienced relatively high mortality risk since the late 1990s [10–13]. In addition, our study also elucidated a surprisingly high mortality rate among workers employed in primary industries (agriculture, forestry, and fishing) and mining, especially mortality from external causes including accidents. This remarkable finding highlights the need for health professionals and policymakers to devote increase attention and resources to help reduce these mortality inequalities. To achieve a decline in mortality rates among service workers; agriculture, forestry, and fishing workers; and administrative and managerial workers will depend on eliminating these mortality inequalities in Japan.

Service workers in the current study continued to have relatively high mortality rates (by both occupation and industry), a trend which has also been reported in previous Japanese studies [11–13]. Service workers are generally classified as lower non-manual workers, as defined by the Erikson-Goldthorpe-Portocarero scheme [22]. Lower non-manual workers are defined as those with mid-level socioeconomic status, and mortality rates within this group have been reported to lie somewhere between that of upper non-manual and manual workers in European countries [8, 10]. The high mortality rate among service workers in Japan deviates from this pattern, however, a trend that has been observed since the 1980s [13]. One possible explanation relates to the irregular work shifts among service workers [23]. In Japan, the opening hours of service industries such as restaurants, bars, supermarkets, and convenience stores are generally long, and stores that remain open 24 h are fairly common. This custom results in service workers having long shifts and night-shift working schedules, which increases their health risks. One restaurant company in Japan recently announced a change in their current 24-h opening times [24]. They cited insufficient staff as the reason for this change; however, such changes would ease the heavy physical and mental occupational burden among service workers in Japan.

Managers had the third highest mortality rate among the 11 occupations examined in this study, which is similar to previous research where a rapidly increasing mortality among managers and professional workers was observed between 1995 and 2000 [11, 12]. This trend was unique to Japan, somewhat dissimilar to trends observed in European countries [10]. The higher mortality among high-grade managers and professionals than that among workers in other occupations is currently under deliberation in Japan. One possible explanation is that Japanese managers have a heavy job burden owing to the need to engage in both managerial work and field work. Indeed, the percentage of Japanese managers decreased after 2000 [13]. We need to follow the trend carefully in the near future.

Our analysis revealed that male Japanese workers in primary industries (agriculture, forestry, and fishing) and workers in mining (defined as secondary industry according to the Japan Standard Industrial Classification) generally have a relatively high mortality rate, a trend that has actually been observed for decades among Japanese national data [13]. In particular, high mortality from external causes was observed among workers in mining and in agriculture, forestry, and fishing. Although work environments have improved and the physical burden placed on workers has been greatly reduced due to automation in primary industries and mining, our findings nonetheless suggest that health risks from occupational accidents are still relatively high in Japan. Regarding international comparisons, farmers in European countries have been shown to have slightly higher mortality than non-manual workers with higher socioeconomic status, whereas Japanese farmers have clearly higher mortality rates [8, 10]. The socioeconomic status of Japanese farmers may differ from that of European farmers, however, where income is generally guaranteed by the European Union’s Common Agricultural Policy. Workers in primary industries must face periods of unstable income, labor shortages, and a lack of replacement workers because young people perceive these as high-risk and burdensome occupations. Therefore, special attention should be paid for workers in primary industries and mining at the site of occupational health. Further study is needed to disclose why mortality among workers in primary industries remains high in Japan.

It is worth noting that certain limitations may have influenced the generalizability of our current results, particularly that for some occupations and industries; mortality should be interpreted carefully owing to potential selection and/or misclassification biases. First, we analyzed data from the National Vital Statistics using a cross-sectional study design. Population estimates by occupation and industry (denominator) were obtained using National Census data, whereas the number of deaths (numerator) was reported by the family of workers who died, a situation in which family members are asked to provide the worker’s occupation and industry at the time of death. This approach may result in misclassification of occupation and industry, a situation which might then result in both overestimation and underestimation of mortality. In Japan, when an individual loses their job after a serious illness, for example, they are reported as being “unemployed” if they later die. Although such a system follows the rules of vital statistics, some occupation-specific and industry-specific mortality may end up being underestimated.

On the other hand, however, workers whose employment status is described as “self-employed” may be overestimated because they are not “laid off” due to illness. For example, mortality rates among agriculture, forestry, and fishing workers may be overestimated because of their employment status, given that approximately 80% of Japanese farmers were categorized as being self-employed in 2015 [17]. The numerator/denominator bias described above has been discussed in the analysis of vital statistics in Japan and South Korea because both countries have not applied linkage methods (e.g., using social security numbers) between the national census and vital statistics [10, 25]. The implementation of individual linkage between Population Census data and social surveys is clearly required, in order to help avoid this limitation in the future.

Second, the definition of various occupations has naturally changed over time, and some occupations are difficult to categorize using the Japan Standard Occupational Classification (e.g., a person who earns advertising revenue using social networking services, such as “YouTubers”). This situation implies that these occupations are reported as “unclassifiable occupation,” “not stated,” or “unemployment.” Indeed, an increase in missing data regarding statistics for these occupations has been reported [10, 13]. In our data set for 2015, 14.1% and 3.2% of men aged 25 to 64 years were recorded as having either an “unclassifiable occupation” or “not stated” occupation, respectively. Therefore, occupation-specific mortality may be underestimated about 15–20% owing to missing data. To our best knowledge, no studies have provided evidence of a numerator/denominator bias in Japan. Further study is necessary, in addition to discussion of way to improve the survey.

## Conclusion

Overall, this study revealed that many preexisting differences in mortality rates among Japanese male workers, according to occupation and industry, have persisted in 2015. Reducing mortality among service workers; agriculture, forestry, and fishing workers; and administrative and managerial workers requires elimination of these mortality inequalities. This study emphasizes that occupational health plays an important role in reform of the working style in Japan.

## Data Availability

No additional data are available.

## Abbreviations

95% CI: 95% confidence interval
MHLW: Ministry of Health, Labour and Welfare
JSOC: Japan Standard Occupational Classification
JSIC: Japan Standard Industrial Classification
RR: Rate ratio
